# Amino acid mutations K54E and S154P in the neuraminidase attenuate H3N2 canine influenza virus in mice

**DOI:** 10.1099/jgv.0.002223

**Published:** 2026-02-20

**Authors:** Xue Pan, Xiaona Shi, Luxiang Zhao, Dawei Yan, Fan Zhou, Qinfang Liu, Chunxiu Yuan, Bangfeng Xu, Zhifei Zhang, Minghao Yan, Qiaoyang Teng, Zejun Li

**Affiliations:** 1 Shanghai Veterinary Research Institute, Chinese Academy of Agriculture Sciences, Shanghai, PR China; 2 College of Veterinary Medicine, South China Agricultural University, Guangzhou, PR China

**Keywords:** amino acid mutation, H3N2 influenza virus, neuraminidase, virulence

## Abstract

Dogs are considered mixing vessels for influenza viruses, posing a pandemic potential via viral reassortment. Our previous studies indicated that the avian-origin H3N2 canine influenza virus (A/canine/Zhejiang/1/2010, abbreviated C1) is virulent in canine and mice. Furthermore, we found that the HA and NA genes of C1 share a close genetic relationship with an H3N2 avian influenza virus (A/duck/Shanghai/06/2009, abbreviated D6), but they exhibit distinct pathogenicity. However, the understanding mechanisms remain unclear. In the present study, we explored the genetic determinants that contribute to the different pathogenicity between the C1 and D6. By using the reverse genetics approaches, we rescued several single-gene and position-substituted reassortant viruses based on the C1. The replication in Madin–Darby canine kidney cells and pathogenic trial in mice showed that the neuraminidase (NA) gene played a critical role in C1 virulence. Further analysis demonstrated that the K54E and S154P mutations in NA significantly reduced NA enzymatic activity, impairing viral release from infected cells. Consequently, these mutant viruses lost their ability to infect mice. Overall, our findings identify two novel virulence determinants in NA and elucidate the mechanisms behind the distinct pathogenicity between the C1 and D6 in mice. These results may provide some new targets for H3N2 influenza virus vaccines and antiviral drug development.

## Data Summary

The authors confirm that all supporting data, codes and protocols have been provided within the article or through supplementary data files.

## Introduction

Influenza A virus (IAV) is a single-stranded and negative-sense RNA virus belonging to the *Orthomyxoviridae* family. Its genome comprises eight segments encoding at least ten different viral proteins, including the surface proteins, haemagglutinin (HA), neuraminidase (NA) and the membrane ion channel (M2) proteins; the internal proteins, nucleoprotein (NP) and matrix protein (M1); and the polymerase complex comprised of the polymerase basic protein 1 (PB1), polymerase basic protein 2 (PB2) and polymerase acidic protein (PA). Besides, there are two non-structural proteins, NS1 and NS2 [1]. Based on the antigenic differences of HA and NA, IAVs are divided into different subtypes. Globally, from 1 January 2003 to 3 June 2025, the H5N1 IAVs have directly caused 986 cases with 473 deaths. The H7N9 IAVs have directly caused 1,568 human infections with 616 deaths since early 2013 (http://www.who.int/influenza). In addition, the H3N8, H5N6, H7N4, H9N2, H10N3, H10N5 and H10Nx IAVs have caused multiple human infections in several countries [2,6]. Critically, pandemics in 1957 (H2N2), 1968 (H3N2) and 2009 (H1N1), all resulting from IAV reassortment, caused millions of deaths and severe socioeconomic burdens [7,8].

Dogs, as companion animals with close human contact, provide great opportunities for the reassertion of influenza viruses via co-infection in a host, resulting in new strains with pandemic potential [9]. The H3N2 canine influenza virus (CIV) was first isolated from a pet dog in South Korea [10]. Since then, the virus has spread throughout South Korea, China, the USA and Canada [11,12]. The I714S mutation in PB2 enhances mammalian adaptation of H3N2 canine influenza virus by impairing nuclear import efficiency and RNP complex assembly [13]. The mutations (HA2-K82E, HA2-R163K and NA-S18L) critically contributed to the improved replicative capacity of CIV in human cell cultures [14]. Phylogenetic analysis suggested that H3N2 CIV was of avian origin [10,15]. However, little is known about the underlying mechanisms of transmission of H3N2 IAV from avian to canine species.

Our previous studies indicated that the avian-origin H3N2 CIV (A/canine/Zhejiang/1/2010, abbreviated C1) isolated from the lung of a Tibetan mastiff could infect dogs and mice [15]. Furthermore, we found that the HA and NA genes of C1 share a close genetic relationship with an H3N2 avian influenza virus (A/duck/Shanghai/06/2009, abbreviated D6), but they exhibit distinct pathogenicity in mice. However, the mechanisms under this difference in pathogenicity remain unclear. In the present study, we explored the mechanisms underlying C1 cross-species transmission from avian to canine species. Since the surface genes, HA and NA, are two of the most important genes for the pathogenicity of IAVs [16,17], we first rescued two reassortant viruses by replacing either the HA or NA gene of C1 with the corresponding gene from D6. The pathogenic trial in mice revealed that the NA gene played a critical role in viral pathogenicity, and the two specific amino acid mutations in NA significantly contribute to C1 virulence. Finally, we proved that the two specific amino acid mutations would enhance NA enzymatic activity, leading to the release of more progeny viruses and resulting in different pathogenicity in mice.

## Methods

### Cells and viruses

Madin-Darby canine kidney (MDCK) cells and human embryonic kidney cells (HEK-293T) were maintained in Dulbecco’s Modified Eagle’s Medium supplemented with 10% fetal bovine serum. The avian-origin H3N2 CIV (C1, GenBank: JF714155.1) and H3N2 avian influenza virus (D6, supplementary 2) were isolated and provided by the Etiologic Ecology of Animal Influenza and Avian Emerging Viral Disease group of Shanghai Veterinary Research Institute.

### Virus rescue and pathogenicity testing

We cloned the cDNAs of each full-length RNA segment of the C1 and D6 viruses into a viral RNA–mRNA bidirectional expression plasmid (pBD) by using the gene segment-specific primers shown in Table 1. All of the constructs were completely sequenced to ensure the absence of unwanted mutations. By using these plasmids, all related viruses were rescued in HEK 293 T cells according to the eight-plasmid system [18]. We grew the rescued viruses in 10-day-old embryonated specific-pathogen-free (SPF) chicken eggs. Three mice were inoculated intranasally with 10^6^ 50% egg infective doses (EID_50_) of each virus housed separately in an isolator. We successfully rescued rD6, rC1 and NA-based mutant viruses in the backbone of C1, but failed to rescue NA-based mutant viruses in the backbone of D6.

**Table 1.** Primers used for pBD cDNA construction and for introducing mutations into the NA genes of its mutant viruses

Purpose	Primers (5′−3′) Forward	Reverse
PB2 amplification	ccAGCGAAAGCAGGTC	ttAGTAGAAACAAGGTCGTTT
PB1 amplification	CACACAGCTCTTCGGCCAGCGAAAGCAGGCA	CACACAGCTCTTCTATTAGTAGAAACAAGGCATTT'
PA amplification	CCAGCGAAAGCAGGTAC	TTAGTAGAAACAAGGTACTT
HA amplification	CCAGCAAAAGCAGGGG	TTAGTAGAAACAAGGGTGTTTT
NP amplification	CACACAGCTCTTCGGCCAGCAAAAGCAGGGTA	CACACAGCTCTTCTATTAGTAGAAACAAGGGTATTTTT
NA amplification	CACACAGCTCTTCGGCCAGCAAAAGCAGGAGT	CACACAGCTCTTCTATTAGTAGAAACAAGGAGTTTTTT
M amplification	CACACAGCTCTTCTATTAGCAAAAGCAGGTAG	CACACAGCTCTTCGGCCAGTAGAAACAAGGTAGTTTTT
NS amplification	CACACAGCTCTTCTATTAGCAAAAGCAGGGTG	CACACAGCTCTTCGGCCAGTAGAAACAAGGGTGTTTT
rC1-NA-L24M mutation	AGTATTTTCCTC**A**TGCAGATGCCATC	GATGGCAATCTGA**T**GAGGAAACATACT
rC1-NA-K54E mutation	AGTATGCCATGT**G**A**G**CCAATCATAATA	TATTATGATTGG**C**T**C**ACATGGCACTACT
rC1-NA-S154P mutation	ACATGATAGGATC**C**C**C**CATCGAACTC	GAGTTCGATG**G**G**G**GATCCTATCATGT
rC1-NA-G430R mutation	GTTGATGAGGA**A**GGCCACAAGAGACT	AGTCTCTTGTGGCC**T**TCCTCTTATCAAC

Nucleotides that were changed are underlined and in boldface.

### Studies in mice

Five-week-old SPF BLAB/c female mice (Vital River Laboratories, Beijing, China) were used in these studies. To find whether the surface genes of the influenza virus contribute to the different pathogenicity to mice between C1 and D6, eight mice in each group were inoculated intranasally with 10^6^ EID_50_ of the test viruses in a 50 µl volume. Three animals were euthanized on 3 days post-inoculation (d.p.i), and their lung and nasal turbinates were collected and titrated in embryonated chicken eggs. The other five animals were weighed daily to test the body weight change. Histopathology of the lung was done by a biological firm (BOSTER Biological Technology).

To access the pathogenicity of rC1 and its NA mutant viruses, three mice in each group were inoculated intranasally with 10^6^ EID_50_ of the test viruses in a 50 µl volume and euthanized on 3 d.p.i. The lung and nasal turbinate were collected and titrated in embryonated chicken eggs.

### Viral replication in MDCK cells

To evaluate virus growth kinetics, monolayers of MDCK cells were inoculated with viruses at an 10^6^ EID_50_/T25 and were maintained in Opti-MEM (Gibco, Grand Island, NY, USA) containing 0.2 g ml^−1^ l-(tosylamido-2-phenyl) ethyl chloromethyl ketone (TPCK)-treated trypsin (Sigma-Aldrich, St. Louis, MO, USA) at 37 °C. Aliquots of culture supernatant collected from triplicate cultures at various time points (hours post-infection) were immediately frozen at −80 °C until they were used.

### NA activity assay

All viruses were grown in eggs, concentrated by ultracentrifugation at 32,000 r.p.m. for 3 h at 4 °C and further purified by using 30% sucrose. The purified virus was resuspended in PBS (pH 7.2). The protein concentration in purified virus samples was measured by HA titre. Equal amounts of purified viral proteins (HA=2^11^) were serially diluted twofold in buffer, and NA activity was tested according to the instructions of the NA-Fluor^™^ Influenza Neuraminidase Assay Kit (Invitrogen, USA).

### Release of progeny viruses from MDCK cells

To value the ability to release progeny on MDCK of mutant viruses, monolayers of MDCK cells were inoculated with viruses at 10^6^ EID_50_ per well in 6-well plates and were maintained in Opti-MEM (Gibco, Grand Island, NY, USA) without TPCK at 37 °C. Two hours after attachment, *Vibrio cholerae* neuraminidase (25 mu ml^−1^, Sigma-Aldrich, St. Louis, MO) and *Arthrobacter ureafaciens* neuraminidase (10 mu ml^−1^, Sigma-Aldrich, St. Louis, MO) were added for 1 h at 37 ℃ and washed three times using PBS to remove all virus that was on cell surface; then, new cell culture was put in cell. Group A: 12 and 24 h later, culture supernatants were collected, respectively, and TPCK (1 g ml^−1^) was added for 30 min at 37 ℃, and then they were immediately frozen at −80 °C until they were used. Group B: after collecting culture supernatant at 12 and 24 h, new cultures were added, and *V. cholerae* neuraminidase (25 mu ml^−1^) and *A. ureafaciens* neuraminidase (10 mu ml^−1^) were added for 1 h at 37 ℃. After all viruses were released, culture supernatants were collected, respectively, and TPCK (1 g ml^−1^) was added for 30 min at 37 ℃ and then immediately frozen at −80 °C until they were used. EID_50_ of group A and B was tested for virus releasing ratio A(EID_50_)/[A(EID_50_) + B(EID_50_)].

### Western blotting

Protein samples fractionated by SDS-PAGE were transferred to polyvinylidene fluoride membranes (Merck-Millipore). Membranes were blocked with 5% skimmed milk in PBS overnight at 4 ℃ and then incubated with diluted rabbit anti-NP Mab (GTX125989, Gene Tex, USA) and rabbit anti-glyceraldehyde-3-phosphate dehydrogenase (GAPDH) polyclonal antibody (PAb) (GTX100118, Gene Tex, USA). After three washes with PBST on a shaker, the members were incubated with anti-rabbit IgG HRP (GTX213110-01, Gene Tex, USA). The blots were visualized by using a Tanon 4200SF multifunctional chemiluminescence imaging system (Tanon, China). The densities were normalized to those of GAPDH and calculated as the reassortant viruses/rC1 expression ratios by using ImageJ (version 1.53 m).

### Indirect immunofluorescence assay

The inoculated cells were washed twice with PBS. Paraformaldehyde (4%) was added to stabilize the cells and then incubated with rabbit anti-NP Mab (GTX125989, Gene Tex, USA) and later with fluorescence-conjugated goat anti-rabbit immunoglobulin G-FITC (ab6717, Abcam). The results were observed by inverse microscopy (magnification ×40W).

### Statistical analysis

Statistical analysis was performed using GraphPad Prism version 6.0 for Windows (GraphPad Software, San Diego, CA) and SPSS 16 for Windows (SPSS Inc., Chicago, IL). Significant differences were calculated using one-way ANOVA followed by the Tukey test. Symbol (*) denotes differences between two groups (*P<0.05, **P<0.01, ***P<0.001, ****P<0.0001).

## Results

### The NA gene plays a vital role in the different pathogenicity in mice between the C1 and D6

**Fig. 1.**
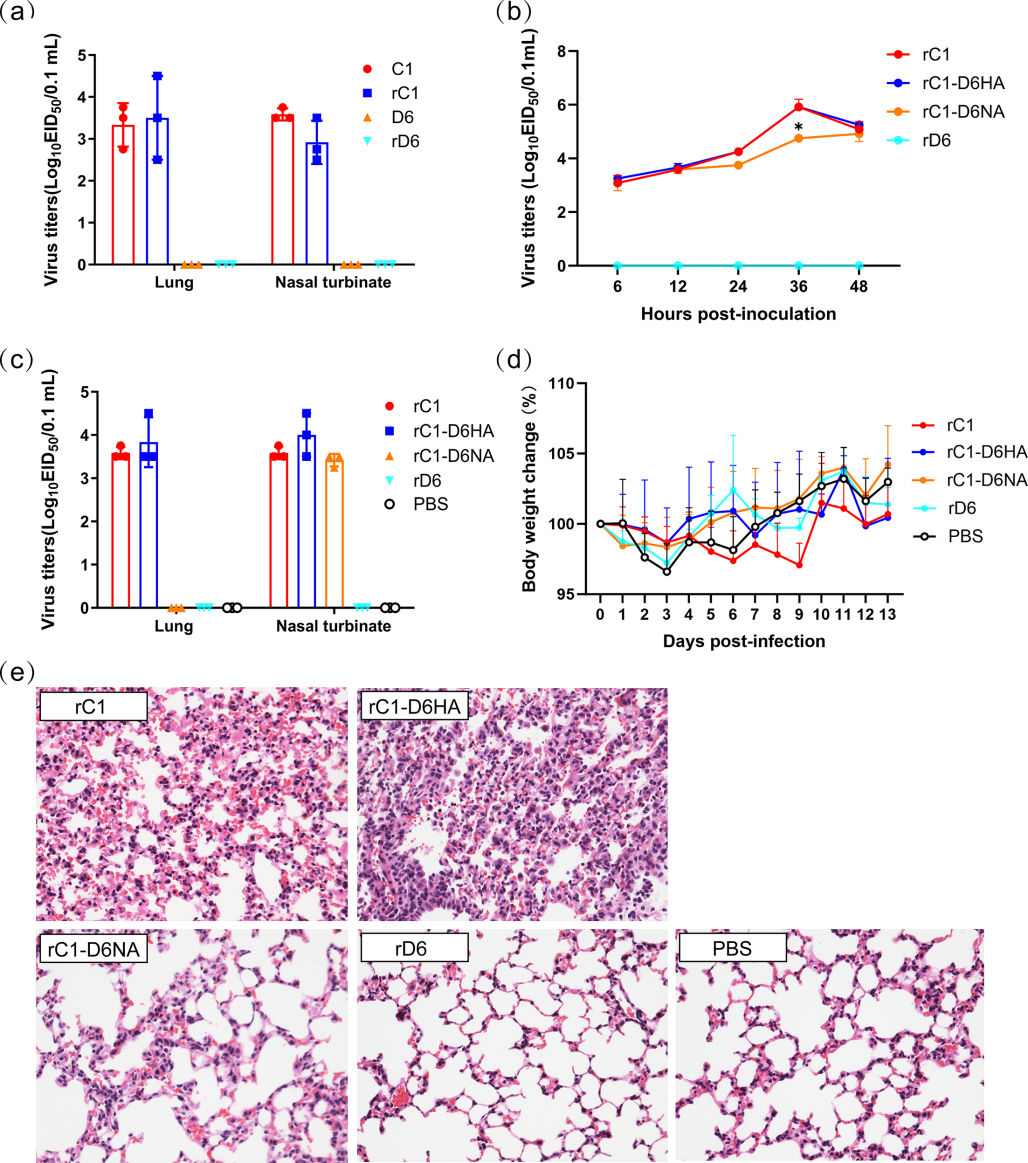
Replication and virulence of C1, D6 and its reassortant viruses in mice. (**a**) Replication of rC1, rD6 and their wild-type strains in mice. Mice (*N*=3/group) were inoculated with 10^6^ EID_50_ of the indicated virus and euthanized on 3 d.p.i. Lungs and nasal turbinate were collected for virus titration in eggs. (**b**) Replication of rC1, rD6 and their reassortant virus *in vitro*. MDCK cells were infected with 10^6^ EID_50_ of the indicated virus. Culture supernatants were harvested at 6, 12, 24, 36 and 48 h.p.i., respectively, and subjected to the EID_50_ assay. Virulence of C1-based reassortant viruses in mice. Mice (*N*=8/group) were inoculated with 10^6^ EID_50_ of the indicated virus, and three of them were euthanized on 3 d.p.i. The lung and nasal turbinate were collected for virus titration in eggs (**c**) and histological study (**e**). The other five animals were used to detect body weight change (**d**).

To identify whether the pathogenicity of the rescued viruses changed compared with their wild-type, we rescued two viruses, called rC1 and rD6, that were the same genome as C1 and D6, respectively, by using reverse genetics approaches. Mice were euthanized at 3 d.p.i. Their lung and nasal turbinate were collected and titrated in embryonated chicken eggs. The results showed that viruses were detected in the lungs and nasal turbinate of mice infected with C1 and rC1. No viruses could be detected in mice infected with D6 and rD6 (Fig. 1a). These results suggest that the rescued viruses maintain the same pathogenicity as their wild-type viruses.

The phylogenetic tree of HA and NA genes showed the C1 and D6 shared a close genetic relationship (Material S1, available in the online Supplementary Material). However, they exhibited distinct pathogenicity. To identify whether the surface proteins HA and NA contribute to the pathogenicity of these viruses in mice, we generated surface protein reassortant viruses by using the same approach as described previously [18]. We used C1 as the backbone to generate two reassortant viruses by changing the HA and NA from D6 to C1, called rC1-D6HA and rC1-D6NA, respectively. The results of growth kinetics in MDCK cells showed that rC1-D6NA got significantly lower viral titres at 36 h post-inoculation (Fig. 1b).

To assess the virulence, we inoculated groups of eight mice intranasally with 10^6.0^ EID_50_ of rC1, rD6, rC1-D6HA or rC1-D6NA. The pathogenicity in three of the BALB/c mice in each group showed that the rC1-D6NA could not replicate in lung. However, the rC1-D6HA could still infect mice in lung and nasal turbinate (Fig. 1c). During the 13 days’ observation in the other five BALB/c mice in each group, we found that there was no significant change in percentage of body weight change in all groups (Fig. 1d). Histological studies showed that, compared with the lung of rC1 or rC1-D6HA inoculated mice that showed great damage, the lung of rC1-D6NA inoculated mice was not impaired as that of rD6 and PBS-inoculated mice (Fig. 1e). These results suggest that NA plays an important role in the different pathogenicity between the C1 and D6.

### The amino acid lysine (K) at position 54 and serine (S) at 154 of NA are critical for the C1 *in vitro* and *in vivo*

**Fig. 2.**
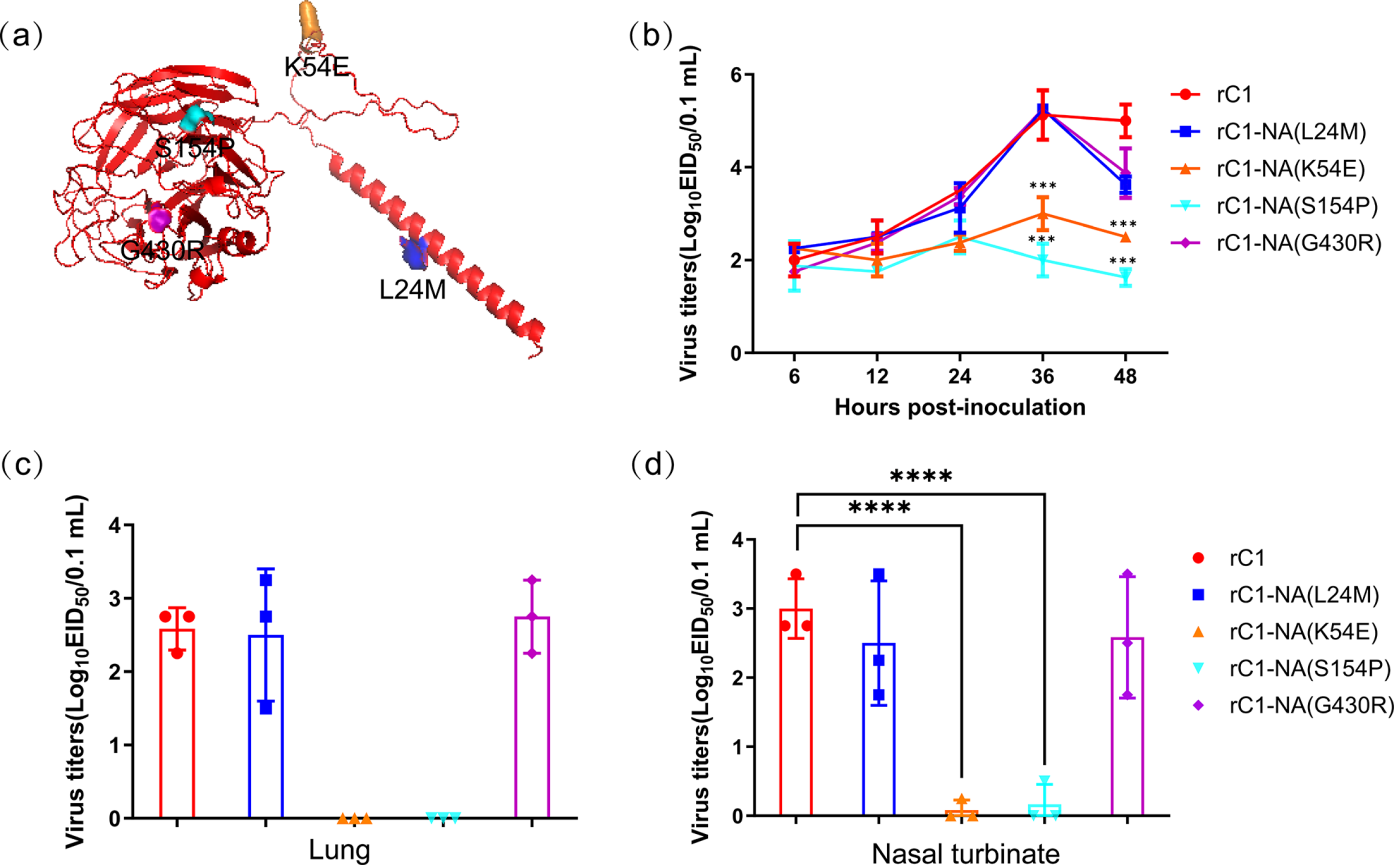
Replication of rC1 and its NA mutant viruses in mice. (**a**) Monomer structure of C1 NA and the amino acid position of NA mutants by using SWISS-MODEL (https://swissmodel.expasy.org/) and AlphaFold3. (**b**) Replication of rC1 and its NA mutant viruses *in vitro*. MDCK cells were infected with 10^6^ EID_50_ of the indicated virus. Culture supernatants were harvested at 6, 12, 24, 36 and 48 h.p.i. and subjected to EID_50_ assay. Virulence of rC1 and its NA mutant viruses in mice. Mice (*N*=3/group) were inoculated with 10^6^ EID_50_ of the indicated virus in a 50 µl volume and euthanized on 3 d.p.i. Lung (**c**) and nasal turbinate (**d**) were collected for virus titration in eggs.

After comparing the conservative region of H3N2 NA proteins from different hosts (human, canine, swine, equine, chicken and duck), downloaded from GenBank, we found four conservative amino acid positions only in NA protein of H3N2 CIVs but not in other H3N2 IAVs, which potentially contributed to the pathogenicity of H3N2 CIVs in mice (Fig. 2a). To determine which amino acid contributes to the pathogenicity of the C1 virus, we generated the four mutants in the C1 background, each of which carried a mutation in NA that was present in the D6 virus at that position. The growth properties of these viruses in MDCK cells showed that the titres of rC1-NA(K54E) and rC1-NA(S154P) viruses had little increase during the whole period, while rC1 and the other two mutants progressively increased and peaked around 10^5^ EID_50_/0.1 ml at 48 hours post-inoculation (h.p.i) in MDCK cells (Fig. 2b).

To make clear their pathogenicity in mice, we infected three mice with 10^6^ EID_50_ of the indicated virus. Their lung and nasal turbinate were collected on 3 d.p.i. for virus titration in eggs. The results showed that the mice infected with mutant viruses rC1-NA(K54E) or rC1-NA(S154P) showed significantly lower viral titres compared with rC1 and other mutant viruses in the lung (Fig. 2c) and nasal turbinate (Fig. 2d). Therefore, 54E and 154P of NA are vital for the pathogenicity of the C1 virus in mice.

### The amino acid mutations K54E and S154P in NA reduce the NA activity

**Fig. 3.**
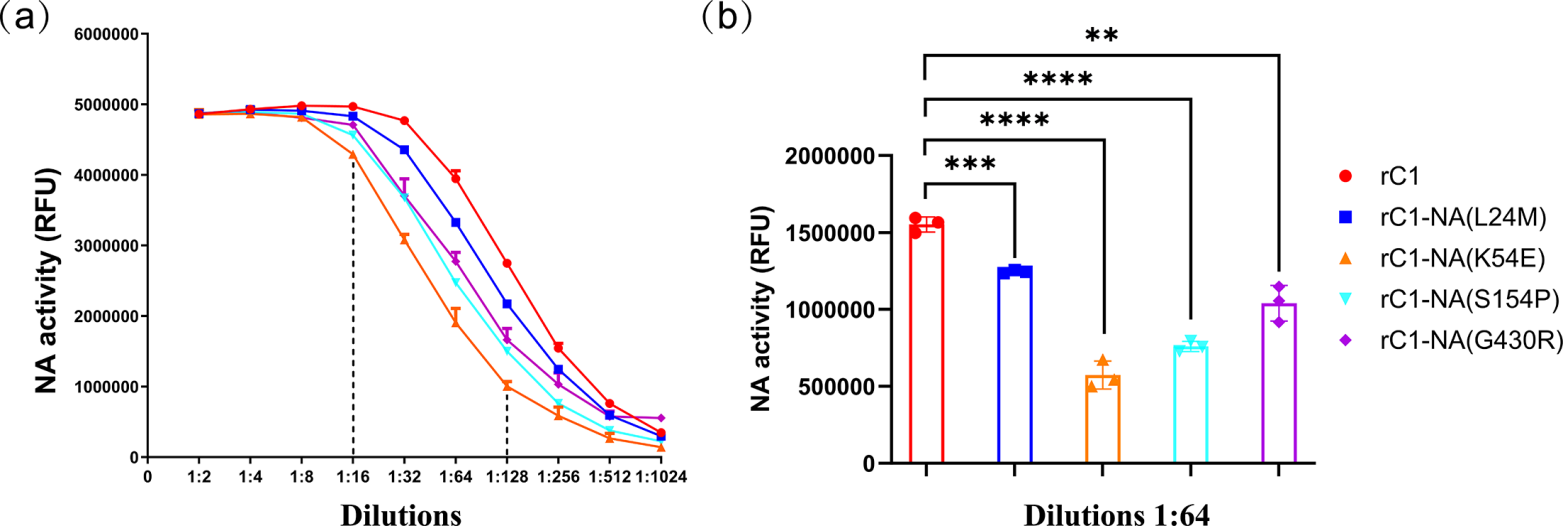
NA activity. The NA activity of purified reassortant viruses was measured by using the NA-Fluor^™^ Influenza Neuraminidase Assay Kit according to the manufacturer’s instructions. (**a**) The NA activity of these purified viruses was detected after serial two-fold dilution. The dilution from 1 : 16 to 1 : 128 showed a linear correlation and was thought to be valid according to the manufacturer’s instructions. (**b**) The dilution 1 : 64 was presented to show the difference in NA activity.

To determine whether the amino acid mutations affect the NA activity, the NA-Fluor^™^ Influenza Neuraminidase Assay Kit was used in the present study according to the manufacturer’s instructions. As shown in Fig. 3a, the results from dilutions 1 : 16 to 1 : 128 showed a linear correlation and were thought to be valid according to the manufacturer’s instructions. The results in the dilution 1 : 64 showed that the NA activity of rC1-NA(K54E) and rC1-NA(S154P) was the lowest and significantly lower than that of rC1, though the NA activity of the other mutant viruses was also significantly lower than that of rC1 (Fig. 3b).

### The amino acid mutations K54E and S154P in NA reduce the release of progeny viruses from MDCK cells

**Fig. 4.**
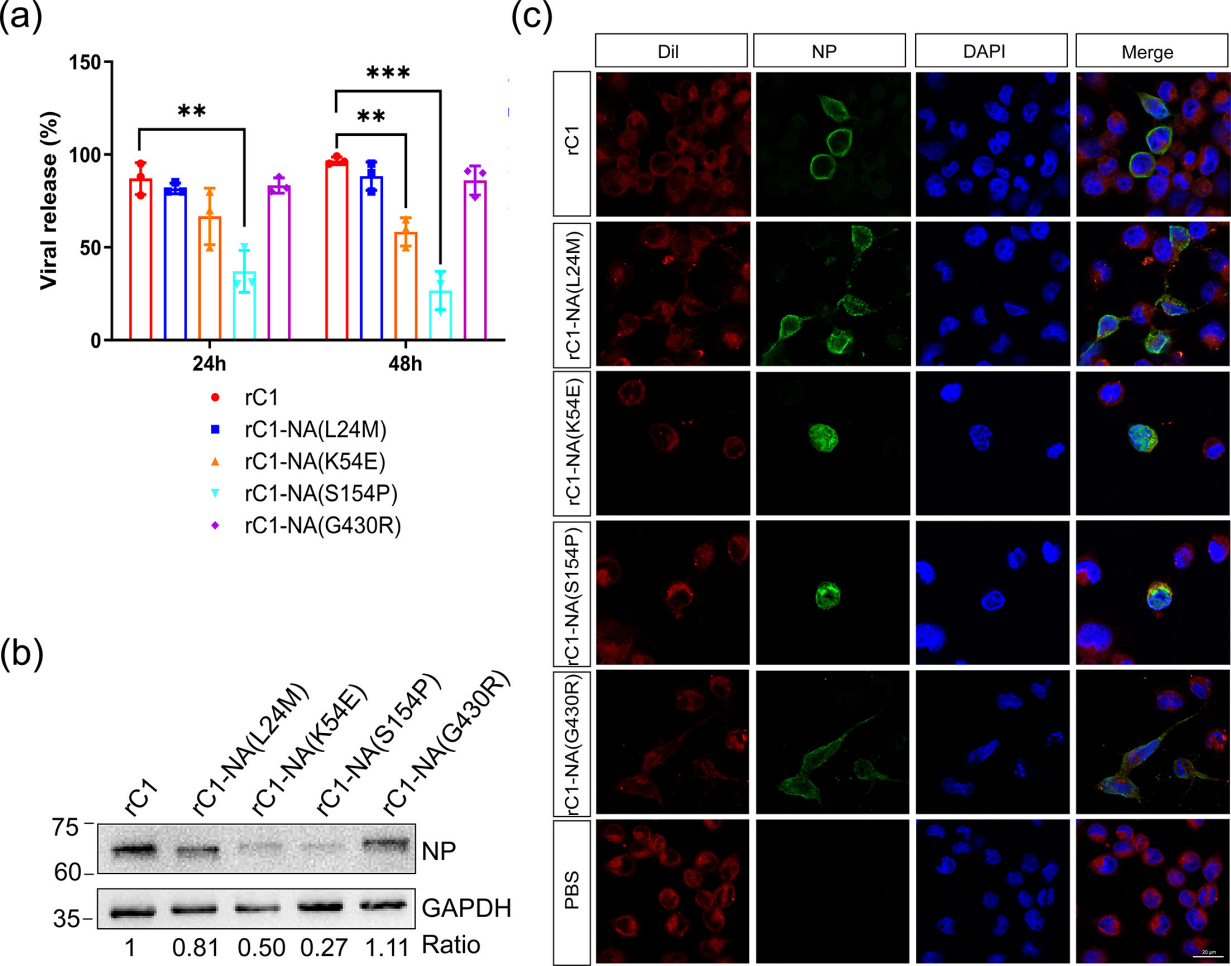
The viral release and location of rC1 and its NA mutant viruses *in vitro*. (**a**) Viral release. MDCK cells were infected with the indicated virus. As a control, the infected MDCK cells were treated with bacterial sialidases for 1 h at 37 °C to release all budded viruses from the cell surface. Virus titres in each supernatant were determined by EID_50_. (**b**) Western blotting. The viral release was detected in the supernatant of virus-infected MDCK cells at 48 h.p.i. and subjected to Western blotting. (**c**) Indirect immunofluorescence assay. The location. MDCK cells were infected with 10^6^ EID_50_ of the indicated virus. After 24 h, the indirect immunofluorescence assay was performed to visualize the location.

Since the function of NA is to release progeny of influenza viruses from infected host cells, we tested the release proportion of progeny viruses from virus-infected MDCK cells by treatment with bacterial sialidases. The results showed that the proportion of released progeny viruses of rC1-NA(S154P) was significantly lower than that of rC1 at 24 and 48 h.p.i. The proportion of released progeny viruses of rC1-NA(K54E) was significantly lower than that of rC1 at 48 h.p.i. (Fig. 4a). Western blotting in Fig. 4b results showed that there were fewer viruses detected in the supernatant of rC1-NA(K54E)- and rC1-NA(S154P)-infected cells, which suggested that the amino acid mutations K54E and S154P in NA reduce the release of progeny viruses from MDCK cells.

The indirect immunofluorescence assay was performed to detect the location of rC1 and its NA mutant viruses. The results showed that the rC1-NA(K54E) and rC1-NA(S154P) were mainly located in the cytoplasm of MDCK cells, indicating impaired viral budding and release. On the other hand, the rC1, rC1-NA(L24M) and rC1-NA(G430R) were mostly located on the surface of cells (Fig. 4c).

## Discussion

Our previous studies showed that D6 (H3N2 AIV) failed to infect mice, but C1 (H3N2 CIV), which was believed to be derived from H3N2 AIV, could infect mice well, leading to lung damage [15]. However, the mechanisms were still unclear. In the present study, we explored the genetic basis of HA and NA proteins of C1 and D6. The data showed that NA was vital for the replication of rC1 in MDCK cells and mice. Furthermore, mutations K54E and S154P in C1 significantly decreased the NA activity *in vitro*. As a result, the reassortant viruses of C1 with K54E or S154P in NA reduced the release of progeny viruses from virus-infected host cells, leading to the failure of replication in the lungs of mice.

Avian influenza viruses can cross the host barrier to infect various mammals, including dogs, cats, tigers, leopards and raccoon dogs. Among them, H3 avian influenza viruses are particularly active, capable of cross-species transmission to humans, horses and dogs, leading to the emergence of distinct influenza virus lineages [9,10]. The avian-origin canine H3N2 influenza virus (H3N2 CIV) can spread rapidly among pet dogs and has undergone natural reassortment with the 2009 H1N1 pandemic virus [19]. Studies have shown that a reassortant virus carrying only the NP and HA gene segments from H3N2 CIV was evaluated in a ferret model, suggesting that H3N2 CIV reassortant viruses may pose a risk to public health [20].

The viral surface glycoproteins HA and NA are important for IAVs’ pathogenicity, adaptation and interspecies transmission [21]. Several key amino acids in HA and NA that influence the viral property have been reported [22,30]. In the present study, we found that mutations K54E and S154P in the NA protein decreased its NA activity. As a consequence, progeny viruses could not be released from infected host cells, and the mutant viruses failed to replicate effectively in mice. However, some different results were observed in previous research. Kristina *et al*. reported that the NA activity of H1N1 IAV could be increased with the mutation K329E in NA in ferry [23]. Besides, Lesly Romero-Beltran *et al*. showed that NA substitution S431P in NA of H1N1pdm09 was more likely to increase NA activity to cleave terminal sialic acid and thus promote viral growth in MDCK cells [24]. A possible explanation for this may be related to different subtypes of IAVs. In addition, the difference in mutation position in NA may also contribute to the difference. Our results in Fig. 1c showed that the rC1D6NA virus replicated in the nasal turbinate but not in lung tissue. This may be because the reduced NA enzymatic activity impairs the release of mature viral particles from the cell membrane, leading to replication predominantly in the nasal cavity (the site of challenge), while limiting sufficient spread to the lungs.

Sialic acids (SAs) serve as cellular entry receptors for influenza viruses and play a crucial role in determining the host tropism of IAVs. In dogs, both SA *α*2,3 and SA *α*2,6 receptors have been detected in goblet cells and subepithelial regions of the nasal mucosa and trachea, though SA α2,6 expression appears relatively weaker [31]. In contrast, avian species predominantly express SA *α*2,3 receptors on respiratory epithelial cells, extending from the nasal turbinate throughout the lungs [32]. The primary function of NA is responsible for the cleavage of the IAV receptor, terminal sialic acids, on cells and extracellular proteins and lipids, thereby facilitating virus release and subsequent infection of new cells [33]. Mutations at some positions in NA can be regarded as a marker of host range and adaptive evolution of IAVs. Stefano Elli *et al*. reported that Y347 played a vital role in NA preference for avian-type substrates. Furthermore, the Y347N mutation in the NA protein facilitates the hydrolysis of mammal-type substrates [16]. Avian-origin N2 NA was introduced into the human population with the I275V amino acid substitution in the NA, leading to the H2N2/1957 pandemic [34]. Our previous research proved that C1 may be derived from D6 by using phylogenetic analyses [15].

The mutations E54K and P154S could increase the NA activity of C1 and promote the replication of C1-based reassortant viruses in mammals, which may contribute to the surveillance of H3N2 IAV in the future. However, some limitations of this study should be considered. Firstly, our results suggest that the NA plays an important role in the different pathogenicity between the C1 and D6 strains. However, besides the surface genes HA and NA, it remains unclear whether the other genes of C1 are also crucial for its replication. Secondly, due to the failure of rescuing the reassortant in the backbone of D6, we have failed to prove that the mutations E54K and P154S in D6 NA would increase the NA activity of D6. There is a very interesting result that the rC1-D6NA maintained infectivity in the nasal turbinate; however, the rC1-NA(K54E) and rC1-NA(S154P) failed to infect the nasal turbinate. It might be due to other amino acids in the NA of C1, which differ from those in D6, affecting the spatial conformation of NA and thereby changing its function.

## Conclusion

In summary, we found that mutations K54E and S154P collectively attenuated the pathogenicity of H3N2 CIV by significantly reducing NA activity, which suggests that 54K and 154S in NA may increase NA activity and promote host preference from avian to mammal. Our study will benefit the surveillance of H3N2 IAV by identifying the mutations 54K and 154S in NA. Furthermore, these findings may provide some new targets for H3N2 IAV vaccines and antiviral drug development.

## Supplementary material

10.1099/jgv.0.002223 Uncited Supplementary Material 1.

## Abbreviations

C1: A/canine/Zhejiang/1/2010
CIV: canine influenza virus
D6: A/duck/Shanghai/06/2009
d.p.i.: days post-inoculation
EID_50_: 50% egg infective doses
GAPDH: glyceraldehyde-3-phosphate dehydrogenase
HA: haemagglutinin
h.p.i.: hours post-inoculation
IAV: influenza A virus
MDCK: Madin–Darby canine kidney
NA: neuraminidase
NP: nucleoprotein
PA: polymerase acidic protein
PB1: polymerase basic protein 1
PB2: polymerase basic protein 2
SAs: sialic acids
SPF: specific-pathogen-free
TPCK: l-(tosylamido-2-phenyl) ethyl chloromethyl ketone
