# Supplementary material for: Amino acid mutations K54E and S154P in the neuraminidase attenuate H3N2 canine influenza virus in mice
Source: J Gen Virol. 2026 Feb 20;107(2):002223. doi: 10.1099/jgv.0.002223 (PMC12923156; doi:10.1099/jgv.0.002223)
Supplement: Uncited Supplementary Material 1. [file jgv-107-02223-s001.pdf]

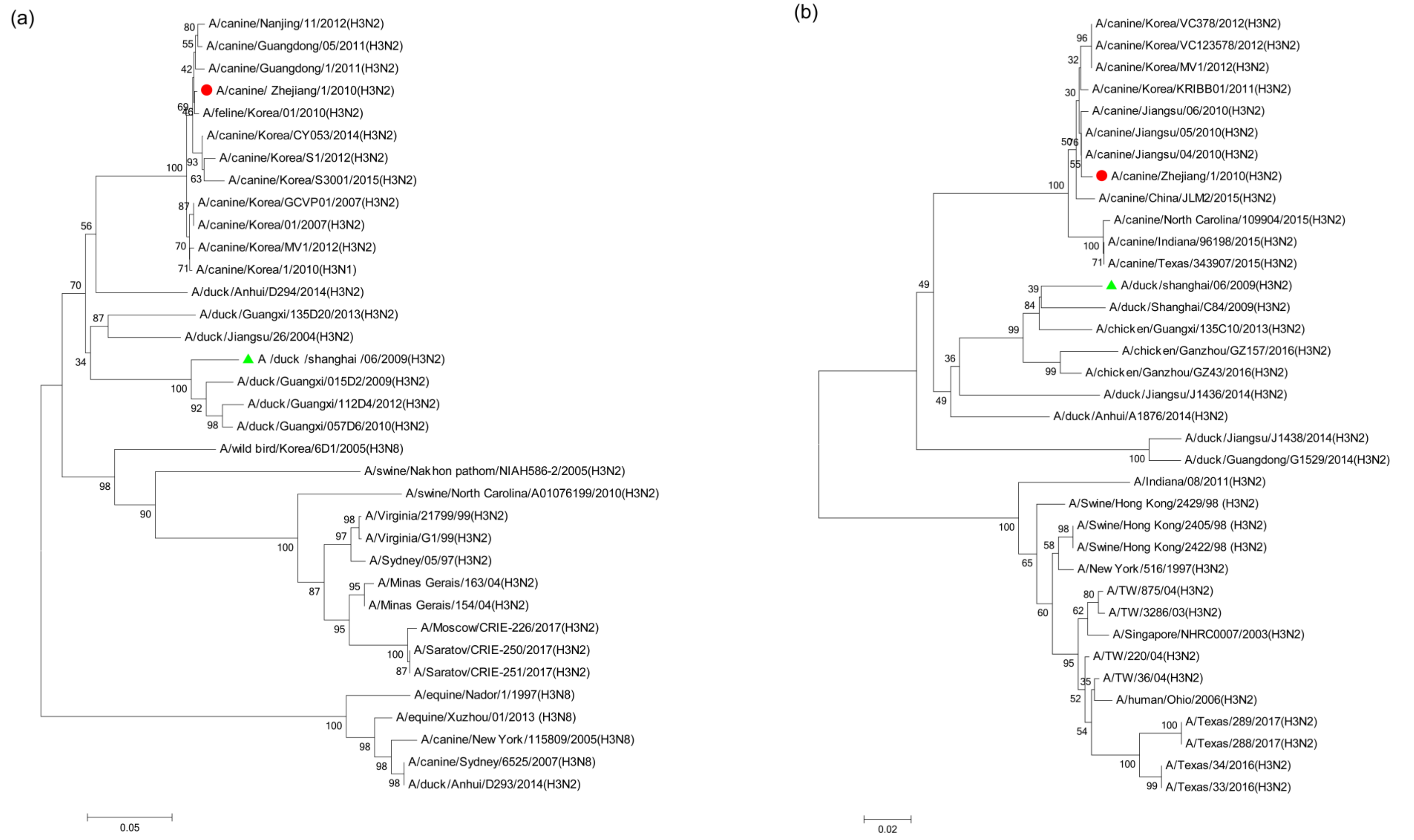

Supplementary 1. The phylogenetic tree of HA (a) and NA (b) of H3N2 influenza viruses.

ATGAATCCAAATCAGAAGATAATTACAATTGGCTCTGTCTCTCTAACCATCGCAATAGTATGTTTCCTCATGCAAATTGCTATTCTCGCAACGAC
TGTAACACTGCATTTCAAGCAAAATGAGTGCAGCATCCCCTCGAACAATCAAGTGGTACCATGTGAGCCAATCATAATAGAGAGGAACATAA
CAGAAATAGTGTATTTAAACAATACTATCATAGAAAAGGGACCTTGTTCTAAGGTGGTAGAATACAGGAATTGGTCGAAACCGCAGTGCCAGA
TTACAGGGTTTGCCCCTTTCTCTAAGGACAATTCAATCAGGCTTTCTGCTGGTGGAGACATTTGGGTAACAAGAGAACCTTATGTGTCATGCAG
TCCAAATAAATGTTATCAATTCGCACTTGGACAGGGAACCACGCTGGACAATAAGCATTCAAACGGCACAATACATGATAGAATCCCCCATCG
AACtCTTTTAATGAACGAATTGGGTATTCCGTTTCATTTGGGAACcAAACAAGTGTGCATAGCATGGTCCAGCTCAAGCTGCCATGATGGAAAA
GCATGGCTGCATGTTTGTGTCACTGGAGATGATAGAAATGCAACTGCTAGTTTCATTTATGATGGGAGGCTTGTTGATAGTATTGGCTCATGGTC
TCAAAACATTCTCAGAACTCAAGAATCAGAATGCGTTTGCATCAATGGAACTTGTGCAGTGGTAATGACTGATGGAAGTGCATCAGGAAGAG
CCGATACCAGAATATTATTCATTAAAGAGGGGAAAATTGCTCATATTAGCCCATTATCAGGGAATGCTCAGCATATAGAGGAATGTTCCTGTTAT
CCCCGGTATCCAGATGTTAGATGTGTTTGCAGAGACAATTGGAAAGGCTCCAATAGGCCCATTATAGATATAAATATGACAGATTATAGCATTGA
TTCCAGCTATGTGTGCTCAGGACTTGTTGGTGACACTCCAAGGAATGATGATGGGTCTAGCAACAGCAATTGCAGAGATCCTAATAATGAGAG
AGGAAACCCAGGAGTGAAAGGATGGGCTTTTGACTACGGAAATGATGTTTGGATGGGAAGAACAATCAGCAAGGACTCACGATCAGGTTATG
AAACTTTCAGAGTTATCAATGGTTGGACCACGGCTAATTCCAAATCGCAGGTAAATAGACAAATCATAGTTGATAATAGTAACTGGTCTGGTTA
CTCTGGCATTTTTTCTGTTGAAAGCAAAAGCTGCATTAATAGGTGTTTTTATGTGGAGTTAATAAGAGGAAGGCCACAGGAGACCAGAGTATGG
TGGACTTCAAACAGTATCGTTGTGTTCTGTGGCACTTCAGGTACCTATGGAACAGGCTCATGGCCTGATGGTGCGAATATCAACTTCATGCCTAT
ATAA

Supplementary 2. The NA ORF sequence of D6.

Virus	Amino acid position in NA			
	24	54	154	430
A/canine/Korea/VC378/2012(H3N2)	L	K	S	G
A/canine/Korea/VC123578/2012(H3N2)	L	K	S	G
A/canine/Korea/MV1/2012(H3N2)	L	K	S	G
A/canine/Korea/KRIBB01/2011(H3N2)	L	K	S	G
A/canine/Jiangsu/06/2010(H3N2)	L	K	S	G
A/canine/Jiangsu/05/2010(H3N2)	L	K	S	G
A/canine/Jiangsu/04/2010(H3N2)	L	K	S	G
A/canine/Zhejiang/1/2010(H3N2)	L	K	S	G
A/canine/China/JLM2/2015(H3N2)	L	K	S	G
A/canine/North Carolina/109904/2015(H3N2)	L	K	S	G
A/canine/lndiana/96198/2015(H3N2)	L	K	S	G
A/canine/Texas/3439071/2015(H3N2)	L	K	S	G
A/duck/shanghai/06/2009(H3N2)	M	E	P	R
A/duck/Shanghai/C84/2009(H3N2)	M	E	P	R
A/chicken/Guangxi/135C10/2013(H3N2)	M	E	P	R
A/chicken/Ganzhou/GZ157/2016(H3N2)	M	E	P	R
A/chicken/Ganzhou/GZ43/2016(H3N2)	M	E	P	R
A/duck/Jiangsu/J1436/2014(H3N2)	M	E	P	R
A/duck/Anhui/A1876/2014(H3N2)	M	E	P	R
A/duck/Jiangsu/J1438/2014(H3N2)	M	E	P	R
A/duck/Guangdong/G1529/2014(H3N2)	M	E	P	R
A/Indiana/08/2011(H3N2)	M	E	P	R
A/Swine/Hong Kong/2429/98 (H3N2)	/	/	/	R
A/Swine/Hong Kong/2405/98 (H3N2)	/	/	/	R
A/Swine/Hong Kong/2422/98 (H3N2)	/	/	/	R
A/New York/516/1997(H3N2)	M	E	P	R
A/TW/875/04(H3N2)	M	E	P	R
A/TW/3286/03(H3N2)	M	E	P	R
A/Singapore/NHRC0007/2003(H3N2)	M	E	P	R
A/TW/220/04(H3N2)	M	E	P	R
A/TW/36/04(H3N2)	M	E	P	R
A/human/Ohio/2006(H3N2)	M	E	P	R
A/Texas/289/2017(H3N2)	M	E	P	R
A/Texas/288/2017(H3N2)	M	E	P	R
A/Texas/34/2016(H3N2)	M	E	P	R
A/Texas/33/2016(H3N2)	M	E	P	R

Supplementary 3. The amino acid position in NA of different H3N2 influenza viruses.

The symbol "/" indicates that the NA gene sequences of these strains are incomplete in NCBI database, with no corresponding amino acid sequences available.
